# Hyoid Cricoid Distance-Based Method Versus Weight‑Based Method for Choosing the Appropriate Size for Classic Laryngeal Mask Airway Selection in Adults

**DOI:** 10.5812/aapm-157335

**Published:** 2025-02-25

**Authors:** Dariush Abtahi, Ardeshir Tajbakhsh, Shahram Sayadi, Mohsen Shojaeian, Gholamali Arab Hesarsheneh, Soudeh Tabashi

**Affiliations:** 1 Imam Hossein General Hospital, Shahid Beheshti University of Medical Sciences, Tehran, Iran; 2 Anesthesia Research Center, Shahid Beheshti University of Medical Sciences, Tehran, Iran; 3 Department of Anaesthesiology, Imam Hossein Hospital, Shahid Beheshti University of Medical Sciences, Tehran, Iran; 4 Department of Anesthesiology, Shohada Tajrish Hospital, Shahid Beheshti University of Medical Sciences, Tehran, Iran; 5 Department of Anesthesiology and Critical Care, Anesthesiology Research Center, Taleghani Hospital, Shahid Beheshti University of Medical Sciences, Tehran, Iran

**Keywords:** Anesthesia, Airway Management, Anthropometry, Laryngeal Masks, Laryngeal Masks/Adverse Effects

## Abstract

**Background:**

Selecting the appropriate sizes for laryngeal mask airways (LMAs) has become a concern for anesthesiologists. Traditionally, size selection has relied on factors like patient weight and gender. Nevertheless, emerging research indicates that incorporating anthropometric data could benefit the identification of the optimal LMA size for individual patients.

**Objectives:**

This randomized controlled trial aims to compare the effectiveness of LMA size selection based on weight against that determined by measuring the hyoid-cricoid distance (HCD).

**Methods:**

A total of 64 patients scheduled for eye surgery under general anesthesia were randomly assigned to two groups, each consisting of 32 participants. In the "W group", the size of the LMA was chosen based on the manufacturer's guidelines, which relied exclusively on the patient's weight. Conversely, in the "HCD group", the selection of LMA size was based on measuring the HCD. We compared postoperative complications and the ease of LMA insertion in these groups.

**Results:**

Data from 28 patients in the W group and 30 in the HCD group were analyzed. The incidence of postoperative complications was comparable between groups, with 16 patients in the W group and 12 in the HCD group experiencing complications (P = 0.1). Additionally, metrics such as the number of attempts, time to insertion, ease of insertion, peak airway pressure, and abnormal curve shape showed no statistically significant differences (P > 0.05).

**Conclusions:**

Based on the findings in this study, the method for selecting LMA size based on HCD did not statistically reduce airway complications nor did it facilitate the insertion process. We recommend conducting larger studies to further investigate this topic.

## 1. Background

The utilization of supraglottic airway devices (SGAs) presents an alternative approach for maintaining airway stability during surgical procedures compared to conventional endotracheal tubes (ETTs) (1). Although SGAs have proven to exhibit high success rates and widespread utilization, it is crucial to acknowledge potential complications. Both ETTs and SGAs must be implemented with precision in terms of appropriate sizing and accurate placement to optimize their functionality and mitigate the occurrence of adverse effects such as throat discomfort and vocal cord paralysis (2).

There exists a broad range of SGAs that serve various purposes: They are classified based on the mechanism of their seal (whether cuffed or uncuffed), the location of their seal (either peri-laryngeal or base-of-tongue), and the type of material they are made of. One of the most common types of SGAs used in operating rooms or available in emergency kits is the laryngeal mask airway (LMA). Choosing the appropriate size of LMA for adults and adolescents could be based on the manufacturer's weight-based model, which recommends number 5 for patients weighing more than 70 kilograms, number 4 for 50 to 70 kilograms, and number 3 for those weighing 30 to 50 kilograms. Some medical practitioners have opted for a sex-based model as opposed to the manufacturer's weight-based model. While manufacturers recommend weight-based criteria for sizing the LMA, this approach may not always be applicable in clinical practice (3, 4).

Previous studies showed that the success rate of LMA insertion when size selection is based on a patient’s weight could be about 77% to 82% (5). It seems that the failure of LMA insertions can be attributed to interindividual anatomical disparities in the peri-laryngeal region. It may be preferable to utilize new sizing criteria in adults that incorporate an evaluation of neck anatomy, rather than relying solely on sex or weight (2). Numerous investigations have been carried out to ascertain the appropriate dimensions of LMA in adults (6). Previous studies have indicated that the regression model's determination of the optimal LMA size surpasses the recommendations provided by manufacturers. These studies consistently suggest that cricoid-mental distance (CMD) is an effective approach for accurately determining the appropriate size of LMA (5, 7-11).

Lee et al. conducted a study that revealed that sonographic measurement of the hyoid-cricoid distance (HCD) exhibited minimal variation during the insertion of the LMA. As HCD and inner cuff length had the least difference in diameter in that study, we hypothesized that HCD could factor in determining the appropriate size of LMA (2).

## 2. Objectives

In this observational study, we assessed the efficacy of using the HCD-based method and manufacturers' recommendations to choose the size of classic LMA for adults and determined its relationship with ventilation efficacy, success rate, and rate of complications.

## 3. Methods

This prospective randomized controlled study was conducted between January and April 2022, with ethics approval from the Research Ethics Committees of Shahid Beheshti University of Medical Sciences (IR.SBMU.MSP.REC.1402.359). After obtaining written consent, 100 adult patients (aged 18 to 65 years) with ASA physical status I and II undergoing elective ophthalmic surgery under general anesthesia, who were planned to have LMA for airway management, were included.

The sample size for this study was determined using statistical calculations informed by prior research (12). A formula for sample size estimation was utilized, incorporating a 95% confidence level and 90% statistical power. Techniques such as the normal approximation method for estimating proportions were applied. Based on this analysis, it was concluded that at least 32 participants per group would be required to effectively evaluate outcomes like dysphagia, sore throat, and mucosal injury associated with LMA insertion.

**Figure 1.**
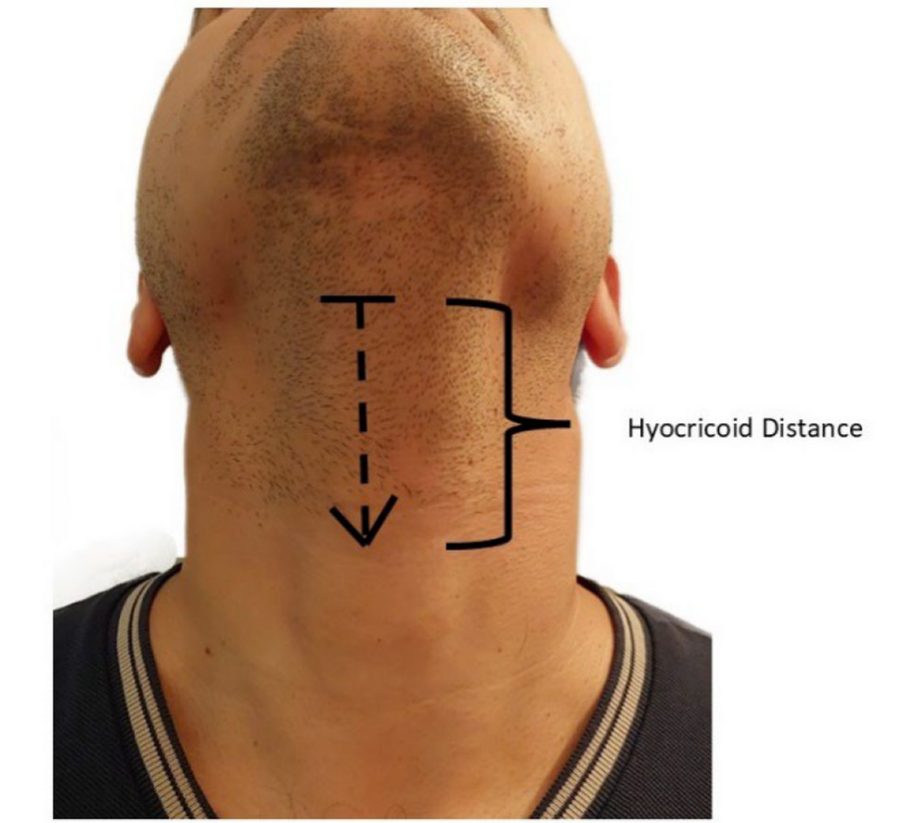
Relative positions of the hyoid bone, cricoid cartilages, and hyoid cricoid distance

All enrolled patients were randomly divided into two groups: The "W group" and the "HCD group", based on the methods of LMA size selection using weight and HCD. In the W group, the size chosen according to the manufacturer's instructions was number 3 for weight less than 50 kg, number 4 for weight between 50 and 70 kg, and number 5 for weight more than 70 kg. The HCD was defined as the linear distance between the inferior border of the hyoid bone and the upper border of the cricoid cartilage when the patient’s neck is in full extension. Measurements were done using fabric rulers (Figure 1). In the HCD-based group, the optimal size was selected as follows: Laryngeal mask airways number 3 for HCD distance < 5.5 cm, number 4 for HCD distance between 5.5 and 6.5 cm, and number 5 for HCD distance ≥ 6.5 cm (13).

Randomization was performed via a random number generator program by the statistical analysis system (SAS Institute Inc., US). Group allocation was performed by a nurse who was blinded to the study method.

All patients were evaluated one day before surgery. Patients were instructed to have nothing by mouth (NPO) for at least 8 hours for solid food and 2 hours for clear liquids before the induction of anesthesia. Patients were in the supine position and were continuously monitored by electrocardiography, non-invasive blood pressure, pulse oximetry, and capnography during surgery. The HCD distance was measured before the induction of anesthesia (Figure 1). Age, sex, weight, height, and past medical history were also recorded for all patients. Patients with limited neck movement or limited mouth opening were not included in this study.

Patients were anesthetized with 1 - 2.5 mg/kg propofol, 2 µg/kg fentanyl, and 0.2 mg/kg atracurium. After loss of consciousness, manual face mask ventilation was established. Once the conditions were sufficiently satisfied, a classic LMA was inserted. The LMA cuff was inflated until appropriate thoracic movement and continuous square end-tidal capnograph traces were observed during mechanical ventilation. If ventilation was inadequate, the operator was allowed to reinsert the LMA with the same size. A maximum of three attempts was performed before insertion was considered a failure. In such cases, the airway was secured with other types of SGAs or an ETT, and the patient was excluded from the study.

After successful insertion, the airway device was fixed with a bandage. The ventilator setup was applied with a tidal volume of 6 cc/kg and an appropriate frequency to maintain end-tidal CO_2_ between 35 - 40 mmHg. The size of the inserted LMA, peak inspiratory pressure, time to successful insertion, ease of insertion, number of attempts, and the shape of the respiratory curve on the ventilator and capnography were recorded. Time to successful insertion was defined as the interval between the beginning of the procedure and the successful insertion of the device. Four grades were considered in the evaluation of ease of insertion, performed by the provider who inserted the device: Grade 1 (no resistance), grade 2 (mild resistance), grade 3 (moderate resistance), and grade 4 (unable to insert the device).

For maintenance of anesthesia, propofol infusion at 100 - 200 µg/kg/min was used. After extubation, patients were transferred to the post-anesthesia care unit (PACU). Postoperative complications were recorded by a blinded research nurse in the PACU after 2 hours, including dry throat, dysphagia, sore throat, hoarseness, and blood presence on the device. In our study, postoperative complications were the primary outcome, and the efficacy of LMA insertion in HCD-based size selection was the secondary outcome.

The distribution of the data was determined using the Kolmogorov–Smirnov analysis. Statistical differences between the two groups were analyzed by the chi-square test for categorical variables, Student's *t*-test for continuous variables, and Mann-Whitney U test for variables on an ordinal scale. A P-value < 0.05 was considered statistically significant. Data analysis was performed using SPSS Inc., Chicago, IL, version 22.0.

## 4. Results

**Figure 2.**
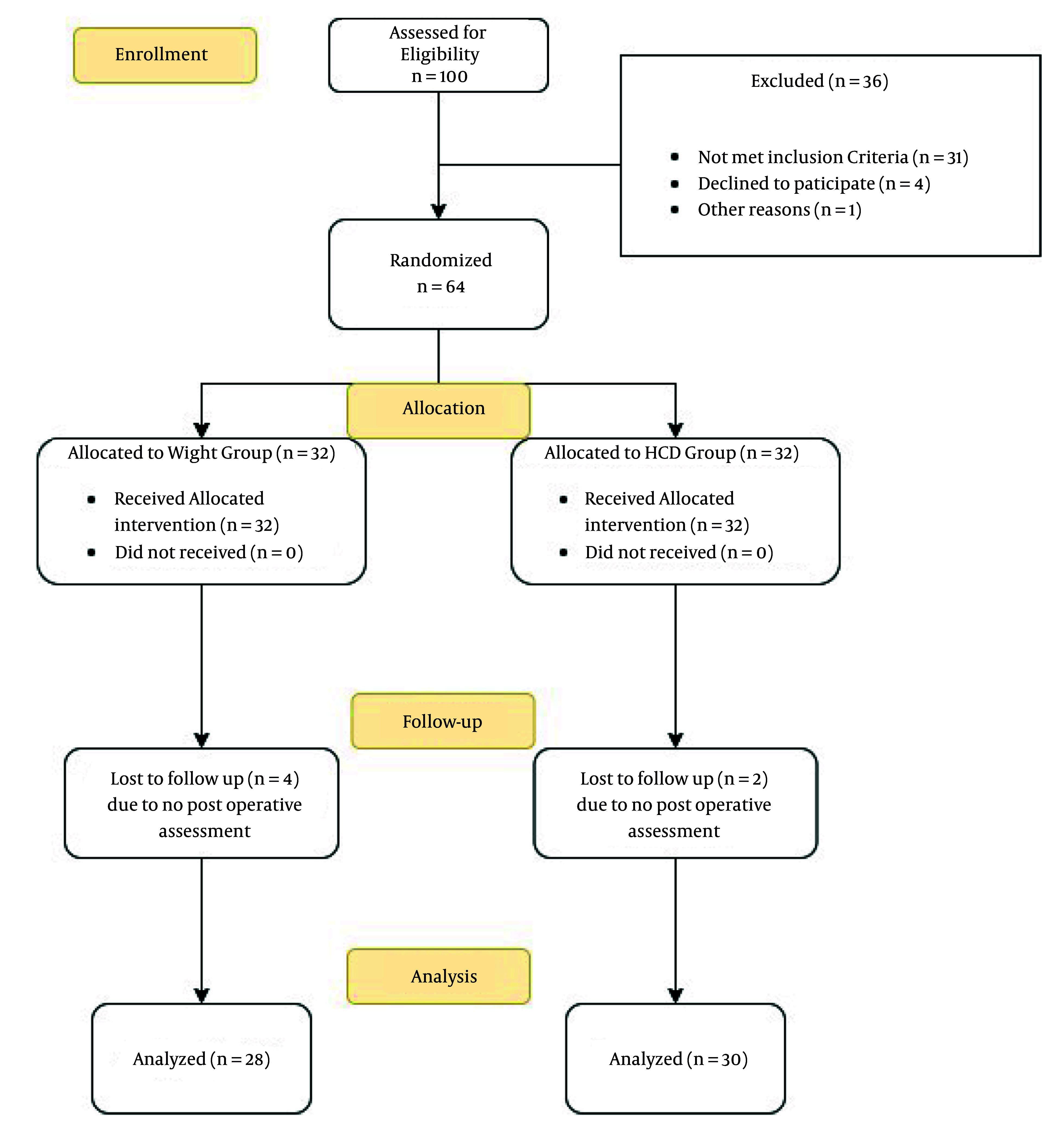
Consort diagram

After screening, 64 patients were enrolled in the study and allocated into two groups of 32 patients each, using the weight-based method and the HCD-based method for size selection of classic LMA. Finally, the data of 28 patients in the W group and 30 patients in the HCD group were analyzed (Figure 2). All demographic characteristics and the duration of anesthesia did not show any significant difference between the two groups. Patients' past medical history was also statistically similar in the W group and HCD group (Table 1). 

**Table 1.** Demographic Characteristics and Past Medical History ^a^

Variables	W Group ^b^	HCD Group ^c^	P-Value
**Age ** ^**d**^	51.7 ± 13.9	53.6 ± 17.1	0.21
**Gender (female/male)**	11/17	11/19	0.69
**Weight ** ^**d**^	66.9 ± 11.5	73.8 ± 11.9	0.07
**Height ** ^**d**^	168.2 ± 10.5	172.1 ± 10.2	0.16
**Mallampati score**	1.3 ± 0.5	1.5 ± 0.7	0.15
**Duration of anesthesia ** ^**d**^	69.8 ± 25.8	67.5 ± 19.9	0.7
**Diabetes**	7	9	NS
**Hypertension**	5	6	NS
**Ischemic heart disease**	2	1	NS
**Asthma**	2	1	NS
**Smoking **	2	1	NS

Abbreviations: HCD, hyoid-cricoid distance; NS, not significant.

^a^ Values are expressed as mean ± SD.

^b^ W group is a weight-based group.

^c^ HCD group is a hyoid-cricoid group.

^d^ These variables are continuous and others are categorical.

In the weight group, 16 patients, and in the HCD group, 12 patients experienced at least one of the postoperative complications, including dry throat, dysphagia, sore throat, hoarseness, and blood presence on the device in the PACU. Although higher in the weight group, the difference was not significant (P = 0.1). The number of attempts to insert the LMA was higher in the weight group but was statistically similar (P = 0.7). Effective airway establishment lasted an average of 10.5 ± 8.8 seconds in the weight group and 8.0 ± 7.4 seconds in the HCD group, which did not show any significant difference (P = 0.2). Size selection of LMA according to HCD did not make the insertion easier than the weight-based method (P = 0.4). Peak airway pressure during anesthesia was the same in both groups (15.4 ± 4.3 vs 15.4 ± 3.7, P = 0.9). The incidence of abnormal respiratory curves on the ventilator was higher in the weight-based method, with a P-value of 0.04. Results of primary and secondary outcomes are summarized in Table 2. 

**Table 2.** Primary and Secondary Outcomes ^a^

Variables	W ^b^	HCD ^c^	P-Value
**Try**	1.20 ± 0.5	1.1 ± 0.5	0.7
**Time to insertion**	10.5 ± 8.8	8.0 ± 7.4	0.2
**Ease of insertion**	1.8 ± 1.1	1.7 ± 0.8	0.4
**Peak airway pressure**	15.4 ± 4.3	15.4 ± 3.7	0.9
**Abnormal curve shape**	6	3	0.04
**Complications **	16	12	0.1

Abbreviation: HCD, hyoid-cricoid distance.

^a^ Values are expressed as mean ± SD.

^b^ W group is weight-based group.

^c^ HCD group is hyoid-cricoid group.

## 5. Discussion

The selection of an appropriate size for LMA holds great significance in ensuring its safe and effective utilization. This study demonstrates that size selection of classic LMA according to HCD did not significantly decrease postoperative complications compared to the manufacturer size selection method based on weight. While the HCD group exhibited a reduced frequency of complications, this difference was not statistically significant. In this study, we defined postoperative airway complications as sore throat, dysphagia, or blood on the LMA upon exertion. This classification was informed by previous research comparing various complication outcomes, which may explain the absence of significant differences observed between the two groups (14).

As a secondary outcome, we found that HCD-based size selection did not facilitate the insertion of the classic LMA. One of the variables assessed in this study was the ease of insertion from the perspective of the anesthesiologist. A single anesthesiologist with moderate experience performed the LMA insertions. After each insertion, a four-point Likert scale was utilized to categorize the ease of insertion as easy, moderately easy, moderately difficult, or difficult. The results of this classification were compared between the two groups, revealing no significant differences. One potential reason for this lack of significance could be that the results may pertain only to anesthesiologists with moderate experience, while variations might exist for nurse anesthetists, anesthesia residents, newly graduated anesthesiologists, or those with extensive experience.

In the operating room, the size of classic LMA is routinely chosen according to patients’ weight or sex. However, previous studies questioned the correlation between weight and larynx size. As an alternative, laryngeal anatomical and radiological distances were suggested (8, 15-17). In 2019, Zhu et al. evaluated CMD in comparison with weight for choosing LMA size. They found that CMD could be an alternative criterion for optimal size selection of LMA due to increased oropharyngeal leak pressure (OLP) and success rate. The OLP was defined as the maximum airway pressure at the time of hearing the noise of gas leakage (5). In other studies, sonographic measurement of hyomental and HCD had a more successful rate than the weight-based method (2, 13).

In our study, HCD measurement by fabric ruler in the preoperative examination was evaluated. Postoperative morbidity, defined as the incidence of any dry throat, dysphagia, sore throat, hoarseness, and blood presence on the device, was our primary outcome to evaluate the adequacy of HCD as a criterion for size selection. According to our results on 64 patients, regardless of any effects on intraoperative factors, postoperative complications were lower in the HCD group, but this was not statistically significant. The reason could be the sample size. It also shows that using this anatomical measurement ultimately could not decrease the complications, which is the aim of any anesthesiologist.

Ease of LMA insertion was the same in both groups, unlike other similar studies (2, 5, 13). As in our previous results, the HCD-based method for LMA size selection resulted in fewer attempts, less time of insertion, lower peak airway pressure, and fewer abnormal curve shapes during surgery. However, none of these differences were statistically significant. Evaluating these factors with a larger sample size could confirm or reject our results.

In light of the potential absence of a ruler within operating rooms, the process of measuring and selecting the appropriate size may prove to be labor-intensive. One practical implication for anthropometric measures in daily situations is predicting distances with finger breadths. In individuals of normal weight and height, each finger breadth measures approximately 2.3 to 2.5 centimeters (18, 19). Consequently, by substituting the numerical values in this article with those of finger breadths, one may deduce that if HCD is less than two fingers, a size number 3 is deemed appropriate; if it falls between two to two and a half fingers, a size number 4 should be selected, and for measurements exceeding two and a half fingers, a size number 5 is advisable. However, it is recommended to measure one’s breadths for enhanced accuracy in the approach. We recommend this approach because utilizing sonographic data may not always be feasible in every operating room. Therefore, relying on consumption patterns based on sonographic findings and integrating these into daily practice could provide an effective solution in the current era.

Given the lack of multiple previous studies defining anatomical thresholds, repetition of the study with a larger sample size is suggested. Concordance with sonographic measurements could also help to find better criteria for selecting LMA size.

### 5.1. Conclusions

Our study demonstrates that size selection of classic LMA results in the same complication rates in both the weight-based method and the HCD-based method. The procedure of LMA insertion became easier in the HCD-based method.

## Data Availability

The dataset presented in the study is available on request from the corresponding author during submission or after publication. The data are not publicly available due to ethics.

## Clinical Trial Registration Code:

IRCT20231022059807N1.

## References

[1] Menna C, Fiorelli S, Massullo D, Ibrahim M, Rocco M, Rendina EA (2021). Laryngeal mask versus endotracheal tube for airway management in tracheal surgery: a case-control matching analysis and review of the current literature.. Interact Cardiovasc Thorac Surg..

[2] Lee SM, Wojtczak JA, Cattano D (2017). Ultrasound comparison of external and internal neck anatomy with the LMA Unique.. J Ultrason..

[3] Ren Y, Cao C, Liang X, Ju Z, Zhang L, Cui X (2021). Validation of manufacturers' laryngeal mask airway size selection standard: a large retrospective study.. Ann Transl Med..

[4] Guo Z, Wang Z, Ji W (2023). Selection of classic laryngeal mask airway size based on ideal body mass in patients with low body mass index: a randomized trial.. Nan Fang yi ke da xue xue bao= J Southern Med Univ..

[5] Zhu Y, Shen W, Lin Y, Huang T, Xie L, Yang Y (2019). Cricoid-mental distance-based versus weight-based criteria for size selection of classic laryngeal mask airway in adults: a randomized controlled study.. J Clin Monit Comput..

[6] Aghadavoudi O, Shetabi H, Saryazdi H, Babayi S (2022). Assessment of neck characteristics for laryngeal mask airway size selection in patients who underwent an elective ocular surgery; A cross-sectional study.. Bulletin Emergency Trauma..

[7] Asai T, Howell TK, Koga K, Morris S (1998). Appropriate size and inflation of the laryngeal mask airway.. Br J Anaesth..

[8] Avidan A, Eden A, Reider E, Weissman C, Levin PD (2015). Multicentre validation of manufacturers' weight-based recommendations for laryngeal mask airway size choice in anaesthetic practice: A retrospective analysis of 20,893 cases.. Eur J Anaesthesiol..

[9] Berry AM, Brimacombe JR, McManus KF, Goldblatt M (1998). An evaluation of the factors influencing selection of the optimal size of laryngeal mask airway in normal adults.. Anaesthesia..

[10] Brimacombe J, Keller C (1999). Laryngeal mask airway size selection in males and females: ease of insertion, oropharyngeal leak pressure, pharyngeal mucosal pressures and anatomical position.. Br J Anaesth..

[11] Cattano D, Van Zundert T, Wojtczak J, Cai C, Callender R, El Marjiya S (2014). A new method to test concordance between extraglottic airway device dimensions and patient anatomy.. Anesthesiology..

[12] Ghai B, Makkar JK, Bhardwaj N, Wig J (2008). Laryngeal mask airway insertion in children: comparison between rotational, lateral and standard technique.. Paediatr Anaesth..

[13] Wang X, Tang Z, Ma W (2021). Hyomental distance measured by ultrasound for size selection of laryngeal mask airway in female patients: a randomized controlled study.. Res Square..

[14] Hung KC, Wu SC, Hsu CW, Ko CC, Chen JY, Huang PW (2022). Efficacy of laryngeal mask airway against postoperative pharyngolaryngeal complications following thyroid surgery: a systematic review and meta-analysis of randomized controlled studies.. Sci Rep..

[15] Rao AS, Yew AE, Inbasegaran K (2003). Optimal size selection of laryngeal mask airway in Malaysian female adult population.. Med J Malaysia..

[16] Wang J, Shi X, Xu T, Wang G (2018). Predictive risk factors of failed laryngeal mask airway insertion at first attempt.. J Int Med Res..

[17] Cattano D, Van Zundert T, Wojtczak J (2019). Laryngeal mask airway and the enigma of anatomical sizing.. J Clin Monit Comput..

[18] Habib SR, Kamal NN (2010). Stature estimation from hand and phalanges lengths of Egyptians.. J Forensic Leg Med..

[19] Agrawal J, Raichandani L, Kataria SK, Raichandani S (2013). Estimation of Stature from Hand Length and Length of Phalanges.. J Evol Med Dent Sci..

